# Anticoagulation in Atrial Fibrillation With Valvular Heart Disease

**DOI:** 10.1161/JAHA.124.038736

**Published:** 2025-02-14

**Authors:** Ana Catarina Fonseca, Claúdia Jorge

**Affiliations:** ^1^ Department of Neurology Stroke Unit Hospital de Santa Maria Lisbon Portugal; ^2^ Institute of Pharmacology and Neurosciences Centro de Estudos Egas Moniz Faculdade de Medicina Universidade de Lisboa Lisbon Portugal; ^3^ Department of Heart and Vessels Hospital de Santa Maria Faculdade de Medicina Universidade de Lisboa Lisbon Portugal

**Keywords:** anticoagulants, atrial fibrillation, blood coagulation, Editorials, health services, heart valve diseases, Atrial Fibrillation, Cerebrovascular Disease/Stroke, Valvular Heart Disease, Anticoagulants

When direct oral anticoagulants (DOACs) were developed, they were meant to entirely substitute vitamin K antagonists (VKAs) in the prevention of thromboembolism and stroke in patients with atrial fibrillation (AF). Many considered that the time had come to put an end to VKAs, which had been approved as a medication to treat thrombi in humans by the Food and Drug Administration in 1954. Indeed, when compared with VKAs, DOACs had several advantages: a better pharmacodynamic profile with subsequently less interactions with food, alcohol, and drugs; rapid onset; a fixed dosing; and no need for lifelong coagulation monitoring.

The results of the randomized clinical trials (RCTs) that compared DOACs with VKAs for the prevention of thromboembolism in patients defined as non– valvular heart disease (VHD) AF showed that DOACs were at least noninferior regarding efficacy, with 50% lower risk of intracranial hemorrhages in the meta‐analyses of the major RCTs, which further supported the initial expectations.^1^

Pathophysiologically, AF can be caused by different mechanisms that lead to remodeling of the left atria and increase the probability of thrombus formation. Among these mechanisms are VHDs that remain an important cause of AF, namely, in low‐ to middle‐income countries. Globally, at least one third of patients with AF have some degree of VHD. This is a subpopulation of particular interest because, as a group, patients with AF and VHD are known to have a higher prevalence of stroke and systemic thromboembolism than patients with AF without VHD.^2^ Therefore, the indication of DOACs versus warfarin also needed to be evaluated in this subpopulation.

The results of the RE‐ALIGN (Randomized, Phase II Study to Evaluate the Safety and Pharmacokinetics of Oral Dabigatran Etexilate in Patients After Heart Valve Replacement) trial that tested dabigatran versus warfarin in patients with aortic or mitral mechanical heart valves brought some disappointment, however, as the trial had to be stopped early because of an excess of thromboembolic and bleeding events in the dabigatran group, as compared with the warfarin group.^3^ Subsequently, an RCT that compared apixaban with warfarin to prevent valve thrombosis or thromboembolism after implantation of a mechanical aortic valve (PROACT Xa [Prospective Randomized On‐X Anticoagulation Clinical Trial]) similarly had to be stopped early due to an excess of thromboembolism in the apixaban group.^4^ Possible explanations for the increase in thromboembolic complications with DOACs compared with warfarin included inadequate plasma levels of the drug and a mechanism of action that differed from that of warfarin. Authors of the RE‐ALIGN study hypothesized that although the choice of a target trough plasma level of 50 ng/mL of dabigatran was primarily based on data from the RE‐LY (Randomized Evaluation of Long‐Term Anticoagulation Therapy) trial, they could not exclude the possibility that targeting a higher trough level of dabigatran in RE‐ALIGN would have been more effective for the prevention of thromboembolic complications.^3^ Nevertheless, higher dabigatran doses might have led to unacceptably higher bleeding rates. Also, while in patients with AF, thrombi are formed in the left atrial appendage under low flow, and thrombin generation is believed to be triggered by stasis and endothelial dysfunction, in patients with a mechanical heart valve, thrombin generation can be triggered by exposure of blood to the artificial surface of the valve leaflets and sewing ring, which induce activation of the contact pathway of coagulation.^3^ The sewing ring becomes less thrombogenic once endothelial tissue has formed around it. Warfarin is likely to be more effective than dabigatran in this context because it inhibits the activation of both tissue factor–induced coagulation (by inhibiting the synthesis of coagulation factor VII) and contact pathway–induced coagulation (by inhibiting the synthesis of factor IX), as well as inhibiting the synthesis of factor X and thrombin in the common pathway, while dabigatran only inhibits thrombin, and apixaban is a highly selective factor Xa inhibitor.^3^ It has been hypothesized that if contact activation is intense, the resulting thrombin generation may overwhelm local levels of DOACs, which can lead to thrombus formation on the surface of the valve and associated embolic complications.^3^

Patients with rheumatic heart disease–associated AF were included in the INVICTUS (Investigation of Rheumatic AF Treatment Using Vitamin K Antagonists) trial that evaluated the use of rivaroxaban versus VKAs in these patients. Eighty‐two percent of the patients included had a mitral valve area ≤2 cm, which corresponds to moderate to severe mitral stenosis.^5^ In this RCT, which included 4531 patients, VKAs led to a lower rate of composite cardiovascular events and death than rivaroxaban without a higher rate of bleeding.^5^

The results of these RCTs that evaluated the use of DOACs versus VKAs in patients with AF associated with mechanical heart valves and moderate to severe mitral stenosis raised concerns about the possible extrapolation of these data to patients with other types and degrees of VHD associated with AF. Namely, many clinicians were unsure if they could prescribe DOACs in patients with some degree of VHD.^6^, ^7^

Actually, although the initial RCTs that compared VKAs with DOACs in AF were conducted among patients with so‐called “nonvalvular” AF, patients with VHD other than mitral stenosis (mostly moderate and severe) or mechanical heart valves could be included. Consequently, these trials allowed some patients with native VHD to participate. Bioprosthetic valves and valve repair were allowed in the edoxaban (ENGAGE AF [Effective Anticoagulation With Factor Xa Next Generation in Atrial Fibrillation) and apixaban (ARISTOTLE [Apixaban for Reduction in Stroke and Other Thromboembolic Events in Atrial Fibrillation]) trials, and valve repair in the rivaroxaban (ROCKET AF [Rivaroxaban Once Daily Oral Direct Factor Xa Inhibition Compared With Vitamin K Antagonism for Prevention of Stroke and Embolism Trial in Atrial Fibrillation) trial.^6^ Therefore, this distinction between “valvular” and “nonvalvular” AF has been considered by some as confusing and obsolete.^6^

Nonetheless, there are no specific RCTs designed to evaluate the use of DOACs in patients with valvular AF other than moderate to severe rheumatic heart disease or mechanical valve disease. A meta‐analysis from post hoc analysis from the RCTs ARISTOTLE, ENGAGE‐AF‐TIMI‐48 (Effective Anticoagulation with Factor Xa Next Generation in Atrial Fibrillation–Thrombolysis in Myocardial Infarction 48), RE‐LY, and ROCKET‐AF, which included 58 497 patients (29 284 DOAC‐treated and 29 213 warfarin‐treated) with 2018 stroke/systemic embolism, and 3284 major bleeding events showed that DOACs decreased the risk of stroke/systemic embolism compared with warfarin in patients with (hazard ratio [HR], 0.70 [95% CI, 0.58–0.86]) and without VHD (HR, 0.84 [95% CI, 0.75–0.95]).^7^ The summary effects were also similar in the subgroups for the major bleeding outcome (*P* for interaction=0.64) and indicated that no significant difference exists between DOACs and warfarin.^7^ Two systematic reviews of patients with VHD and AF also concluded that DOACs demonstrated efficacy to reduce the risk of stroke and systemic embolism and safety except in patients with AF with moderate to severe mitral stenosis or mechanical valve.^8^, ^9^ Another recent analysis that used real‐world data from a US‐based commercial health care database suggested lower rates of stroke or systemic embolism and bleeding when comparing DOACs and warfarin in patients with AF and VHD.^10^

The work by Dawwas in this issue of the *Journal of the American Heart Association* (*JAHA*) adds more evidence on the safety and effectiveness of DOACs compared with VKAs in patients with AF and VHD other than moderate to severe mitral stenosis or mechanical heart valves.^11^ This retrospective cohort study used a large US administrative health care database, which included 81 667 patients who had AF and VHD and were new users of DOACs and warfarin. VHD in this study was defined as aortic, mitral, pulmonary, and tricuspid valve disease based on inpatient or 2 outpatients’ codes within 1 year. Patients with a bioprosthetic or mechanical heart valve were excluded. The primary effectiveness outcomes were ischemic stroke or systemic embolism and bleeding for safety. In the matched cohort, DOAC use (versus warfarin) was associated with a lower rate of ischemic stroke or systemic embolism (HR, 0.70 [95% CI, 0.61–0.81]) and bleeding (HR, 0.72 [95% CI, 0.65–0.80]). The authors found a lower rate of ischemic stroke or systemic embolism with rivaroxaban (HR, 0.74 [95% CI, 0.62–0.89) and apixaban (HR, 0.62 [95% CI, 0.52–0.74]) but not dabigatran (HR, 0.89 [95% CI, 0.63–1.26]). They also reported a lower rate of bleeding with rivaroxaban (HR, 0.74 [95% CI, 0.62–0.89]), apixaban (HR, 0.60 [95% CI, 0.53–0.68]), dabigatran (HR, 0.75 [95% CI, 0.58–0.97]), and edoxaban (HR, 0.21 [95% CI, 0.05–0.83]).^11^

In the absence of specific RCTs addressing this subpopulation of patients with VHD, these results therefore reinforce the data showing that treatment with DOACs is safe and effective in patients with AF with a broad spectrum of VHDs such as mitral regurgitation, bioprosthetic valves (including mitral), aortic stenosis, tricuspid regurgitation, valve repair, and transcatheter aortic valve implantation^12^ (Figure).

**Figure 1:**
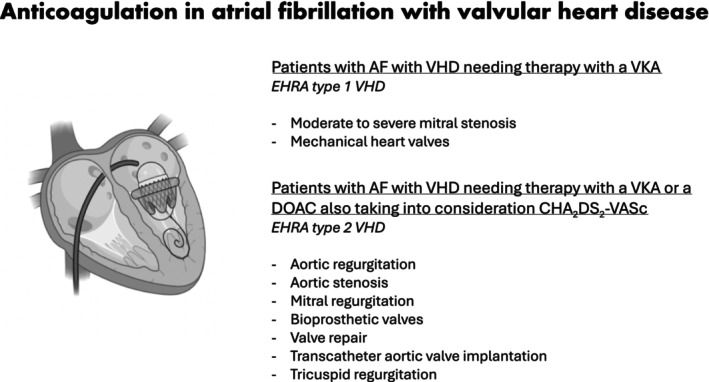
Current indications for treatment with direct oral anticoagulants or vitamin K antagonists in patients with AF and VHD. DOACs indicates direct oral anticoagulants; EHRA, Evaluating Heart Valve, Rheumatic, or Artificial; VHD, valvular heart disease; and VKAs, vitamin K antagonists.

The dichotomization of patients in valvular or nonvalvular AF when addressing treatment with anticoagulants can be misleading for physicians as actually only patients with mechanical heart valves and moderate to severe rheumatic valve disease cannot be currently treated with DOACs. An agreement document from several scientist societies has proposed a functional EHRA (Evaluating Heart Valve, Rheumatic, or Artificial) classification system to direct the choice of oral anticoagulants for the AF and VHD populations.^13^ In this classification, EHRA type 1 VHD refers to patients with AF with VHD needing therapy with a VKA, and EHRA type 2 VHD, refers to AF patients with VHD needing therapy with a VKA or a DOAC, also taking into consideration the CHA_2_DS_2_‐VASc score risk.^13^

Currently included in EHRA type 1 VHD are patients with mechanical heart valves and moderate to severe rheumatic valve disease. In these patients, VKAs still remain, after all these years, as the anticoagulant to be used.^6^, ^12^ We will have to wait and see whether ongoing RCTs such as DAVID‐MS (DAbigatran for Stroke preVention in Atrial Fibrillation in MoDerate or severe mitral stenosis), which is currently evaluating Asian patients with moderate to severe mitral stenosis, will be able further expand the use of DOACs.^14^

## Disclosures

Dr Fonseca reports receiving payment from participation in an Advisory Board for Bayer outside the scope of this article. Dr Jorge has no disclosures to report.
